# Applications of Aramid Fiber-Reinforced Polymer Composites in Civil Engineering: A Review

**DOI:** 10.3390/polym18091102

**Published:** 2026-04-30

**Authors:** Anni Wang, Runping Lan, Qun Chen, Weichen Kong, Haoyu Liu, Qingrui Yue, Xiaogang Liu

**Affiliations:** Research Institute of Urbanization and Urban Safety, School of Future Cities, University of Science and Technology Beijing, Beijing 100083, China

**Keywords:** aramid fiber, aramid fiber-reinforced polymer composites, structural reinforcement, aramid fiber-reinforced polymer composite bars, rock anchor

## Abstract

Aramid fiber is a high-performance fiber with excellent mechanical properties, heat resistance, and corrosion resistance. Its exceptional shear and fatigue properties make it a promising material for civil engineering applications. This study summarizes the basic properties and current development of aramid fiber, as well as the applications of aramid fiber and its composites in civil engineering, including aramid fiber-reinforced composite (AFRP)-concrete/steel composite structures, AFRP rebars, and AFRP rock anchors. The results indicate that the poor interfacial bonding performance between aramid fibers and the resin matrix is the primary bottleneck restricting the application of AFRP composites in civil engineering. Consequently, developing a continuous surface treatment method suitable for industrial-scale production remains a key challenge for the widespread adoption of these composites. Furthermore, in certain specific working conditions and environments—such as seismic retrofitting of rectangular concrete columns, impact/explosion resistance reinforcement, and rock anchoring—AFRPs show the potential to replace traditional inorganic fiber-reinforced polymer composites. However, systematic investigation into the fundamental mechanical properties and long-term service performance of AFRP is still required prior to their practical application.

## 1. Introduction

Fiber-reinforced polymer (FRP) composites have been extensively utilized in the aerospace, automotive, and telecommunications industries due to their lightweight nature, high strength-to-weight ratio, and superior corrosion resistance [1]. Over the past three decades, FRP has found widespread application in structural reinforcement, including strengthening masonry walls, seismic retrofitting of bridges and buildings, repair and reinforcement of concrete and metal structures, as well as the rehabilitation of specialized structures such as chimneys, historical monuments, and offshore platforms [2,3,4]. In recent years, FRP has progressively supplanted conventional steel reinforcement in new construction projects [5,6]. Consequently, the civil engineering sector has emerged as one of the largest global consumers of fiber-reinforced composites [7,8].

FRP composites comprise high-performance fibers as reinforcing materials embedded in a polymeric matrix. The fibers impart exceptional mechanical properties to the composite, with the construction industry primarily employing three types of fibers: E-glass, S-glass, and Z-glass fibers; aramid fibers (e.g., aromatic polyamide, Kevlar 49); and carbon fibers (classified as ultra-high modulus, high modulus, and high strength) [9,10]. Among the matrix materials, thermosetting resins are predominantly used in civil engineering due to their outstanding mechanical performance, with vinyl ester, epoxy, and polyester resins being the most common [11]. Thermosetting resins are cross-linked polymers formed through addition or condensation polymerization, which cure irreversibly under controlled temperature and time conditions. Once cured, they cannot be remelted or dissolved in solvents [12]. Meanwhile, thermoplastic resins are gaining traction in civil engineering applications due to their recyclability and reprocessability, presenting promising prospects for future use [13]. Despite their advantages, carbon fiber-reinforced polymers (CFRPs), the most widely used FRP in civil engineering, exhibit limited shear resistance and inherent brittleness, constraining their applicability in certain structural applications [14]. Similarly, glass fiber-reinforced polymers (GFRPs) suffer from poor fatigue performance and inferior corrosion resistance, significantly restricting their use in new structural systems [15].

Aramid fiber, commercially known as Kevlar fiber, is a high-performance synthetic fiber renowned for its exceptional strength-to-weight ratio, making it indispensable in applications such as ballistic protection (e.g., bulletproof vests), cut-resistant gloves, and aerospace engineering [16]. Initially developed by DuPont in 1965 and commercially introduced in 1971 under the trademark Kevlar, this material was first employed as a reinforcement for tires. Subsequently, its applications expanded significantly, encompassing ballistic armor, high-performance sporting goods, aircraft components, and advanced composite materials in the construction and aviation sectors [17]. Aramid fibers exhibit remarkable mechanical properties, with a specific strength (strength-to-density ratio) exceeding five times that of steel [18]. Additionally, they demonstrate outstanding thermal stability, chemical resistance, and fatigue endurance [19]. Of particular significance, aramid fibers possess superior shear resistance and toughness compared to carbon fibers, rendering them a viable alternative in select engineering applications where brittleness and shear failure are critical concerns. However, the poor moisture and ultraviolet (UV) resistance of aramid fibers are issues that must be carefully addressed before their application in civil engineering.

Aramid fiber-reinforced polymer (AFRP) composites have experienced rapid growth in application, establishing themselves as an essential class of advanced engineering materials. This paper presents a systematic review of: (1) the fundamental chemical composition and physical characteristics of aramid fibers; (2) the basic mechanical properties and durability performance of AFRP composites; and (3) recent advancements in civil engineering applications. Furthermore, the study critically examines current application limitations and proposes potential solutions, with the aim of elucidating future development directions and application prospects for AFRP composites in civil engineering.

## 2. Composition and Properties of Aramid Fiber and Its Reinforced Polymer Composites

### 2.1. Chemical Composition, Microstructure, and Properties of Aramid Fiber

**Figure 1:**
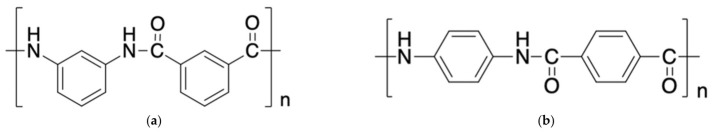
Chemical structure of aramid fibers. (**a**) Meta-aramid fibers (MPDI comes from poly(m-phenylene isophthalamide)); (**b**) Para-aramid fibers (PPTA comes from poly(p-phenylene terephthalamide)).

Aramid fibers are defined as aromatic polyamides in which at least 85% of the amide linkages (—CO—NH—) are directly bonded to two aromatic rings in the polymer backbone [20]. As illustrated in Figure 1, aramid fibers can be classified into meta-aramid and para-aramid according to the positional configuration of these amide linkages. Among them, para-aramid fibers are synthesized via solution (liquid crystalline) spinning of poly(p-phenylene terephthalamide) (PPTA), whose molecular chains exhibit remarkable rigidity due to the conjugation between amide bonds and benzene rings, leading to high internal rotation potential energy and an extended rigid-rod conformation along the fiber axis [21,22]. PPTA comes from poly(p-phenylene terephthalamide). The periodic arrangement of amide groups along the linear backbone promotes the formation of dense and highly oriented intermolecular hydrogen-bonding networks in the lateral direction [23,24], which, together with the rigid aromatic structure, results in exceptional molecular orientation, crystallinity, and intermolecular cohesion [25,26,27]. This highly ordered structure enables para-aramid fibers to achieve outstanding tensile strength, modulus, and thermal stability while also allowing a certain degree of deformation before fracture, thereby contributing to their elongation at break and impact resistance. In contrast, meta-aramid fibers (poly(m-phenylene isophthalamide), MPDI), due to their meta-substituted chain configuration, exhibit lower chain alignment and inferior mechanical properties, and are therefore less commonly used in high-performance structural applications. MPDI comes from poly(m-phenylene isophthalamide).

The performance of para-aramid fibers is intrinsically governed by their hierarchical microstructure. Similar to other fibers produced via coagulation and drawing, their microstructure is commonly described by the skin–core structure model and the lamellar microfiber structure model [28]. In the skin–core model, the core region typically exhibits a highly ordered and crystalline arrangement, whereas the outer skin region is relatively amorphous [29], leading to a radially anisotropic structure that is considered a key factor underlying the superior properties of aromatic polyamide fibers [30]. At the molecular scale, the highly aligned and densely packed chain configuration along the fiber axis further enhances axial load-bearing capacity [31]. Continuous advancements in processing techniques have enabled improved control over molecular orientation and microstructural uniformity, thereby further optimizing the overall performance of para-aramid fibers [32].

Currently, global aramid production is predominantly concentrated in the United States, Japan, and Europe. As shown in Table 1, leading commercial brands include DuPont’s Kevlar^®^ fiber (Wilmington, DE, USA) and Teijin’s Twaron^®^ and Technora^®^ fibers (Matsuyama, Japan). The exceptional mechanical properties of para-aramid fibers stem from their unique structural characteristics. Specifically, para-aramid exhibits a tensile strength of 2.9–5.5 GPa, approximately 5–6 times that of steel wire, along with an elastic modulus of 70–160 GPa, a breaking elongation of ~3%, and notable impact resistance. Leal et al. [33] observed that aramid fibers tend to undergo yielding and kinking under external loads, leading to a more progressive failure mechanism. Moreover, para-aramid fibers are characterized by their low density (1.44–1.45 g/cm^3^), making them significantly lighter than many structural materials. Studies indicate that aramid fiber-reinforced composites can achieve equivalent strength while reducing weight by up to 30% compared to glass fiber composites [34]. Notably, para-aramid does not melt at temperatures around 300 °C and exhibits a high decomposition temperature (~500 °C or above) [35]. Their extremely low coefficient of thermal expansion ensures dimensional stability under extreme thermal conditions [36]. In addition to their mechanical and thermal advantages, aramid fibers exhibit excellent chemical resistance, withstanding exposure to acids, alkalis, and organic solvents, making them suitable for harsh environments [17]. Furthermore, they serve as effective electrical insulators.

The anisotropic nature of aramid fibers significantly influences their fatigue behavior, particularly manifesting in their exceptional tensile-tensile fatigue performance. Experimental investigations have demonstrated that aramid fibers maintain superior fatigue resistance under cyclic loading conditions [40], typically enduring in excess of 10^6^ load cycles without failure at a fatigue stress ratio (R) of 0.5. However, similar to other high-strength organic fibers, aramid fibers demonstrate pronounced yielding behavior under compressive stresses, primarily due to the formation of structural defects known as kink bands [41]. This inherent characteristic results in markedly reduced compressive fatigue performance, particularly under high-strain conditions.

The distinctive molecular configuration endows the aramid fibers with high crystallinity and molecular orientation, accounting for their exceptional tensile strength, modulus, and creep resistance. At low stress levels, aramid fibers demonstrate nonlinear viscoelastic behavior, with creep strains typically reaching magnitudes of 10^−4^ [42]. This deformation arises from reversible alterations in intramolecular bond lengths and angles, resulting in immediate and fully recoverable strain [43]. Under moderate temperature conditions, aramid fibers exhibit notable short-term creep; however, their long-term creep behavior is remarkably stable, with creep strains remaining below 0.1% after one year of loading [44]. While aramid fibers may experience stress rupture under sustained loading, their failure sensitivity under such conditions is substantially lower than that of glass fibers [45]. Within typical service stress ranges, creep deformation in aramid fibers remains minimal and generally inconsequential for long-term structural performance. However, when approaching the material’s yield point or under prolonged high-stress conditions, creep effects may become more pronounced. The creep characteristics of aramid fibers exhibit significant temperature dependence. Although creep deformation is negligible at ambient temperatures, it becomes increasingly substantial when temperatures exceed 150 °C [46]. Consequently, careful evaluation of creep behavior is essential when employing aramid fibers in elevated temperature applications.

Aramid fibers exhibit limited resistance to UV aging, leading to a gradual degradation of their mechanical properties upon prolonged exposure to ultraviolet radiation [47]. Additionally, unlike inorganic fibers, aramid fibers are highly hygroscopic. For instance, at 60% relative humidity, Kevlar 49 exhibits a moisture absorption of approximately 4%, whereas Kevlar 149 absorbs only about 1.5%. This difference reflects variations in their microstructural features, such as crystallinity and chain packing density, which influence moisture uptake and consequently affect their mechanical performance and environmental durability [48]. Despite their susceptibility to moisture and UV degradation, aramid fibers maintain high chemical stability and are largely inert to most organic solvents [49]. However, exposure to strong acids, strong bases, hypochlorites, or elevated temperatures can induce significant degradation in these fibers [50]. Aramid fiber exhibits superior tensile strength compared to glass fiber, greater ductility than carbon fiber, as well as enhanced heat and corrosion resistance. With ongoing advancements in aramid production technology, the material’s performance continues to improve steadily.

### 2.2. Interfacial Bonding Properties Between Aramid Fiber and Resin

In addition to aramid fibers, the performance of AFRP composites is significantly influenced by the type of resin matrix used. Epoxy resins exhibit superior fiber–matrix stress transfer efficiency compared to polyester resins, leading to enhanced shear strength and flexural performance, albeit with reduced impact resistance [51]. In contrast, vinyl ester resins offer a balanced combination of high shear strength and impact resistance [52]. In recent years, thermoplastic resin matrices have gained considerable attention, particularly in short-fiber composites, due to their superior impact resistance and recyclability compared to thermosetting resins [53]. However, thermoplastic-based composites often suffer from inadequate resin infiltration into fiber bundles and weaker fiber–matrix interfacial adhesion. Therefore, when designing AFRPs, selecting an appropriate resin system tailored to the specific performance requirements of the intended application is critical.

The inferior interfacial bonding performance of AFRP composites represents a primary limitation hindering their widespread application in civil engineering. The fiber–matrix interfacial adhesion critically determines the stress transfer efficiency in high-performance FRP, directly influencing both the structural safety and service life of the composite system [54]. However, the intrinsic properties of aramid fibers—including chemical inertness, high crystallinity, and low surface roughness—inherently compromise interfacial bonding with polymer matrices [9]. To address this challenge, numerous surface modification techniques have been investigated, including chemical grafting [55], plasma treatment [56,57], coating deposition [58], and nanostructured surface engineering [59]. Chemical grafting is a modification method that introduces functional molecules or polymer chains onto the fiber surface via covalent bonds (e.g., nitro, amino, carboxyl groups) [55]. Coating deposition involves forming a continuous or discontinuous solid film on the fiber surface via physical or chemical interactions (e.g., dopamine treatment) [56,57]. Nanostructured surface engineering involves constructing nanoscale topographies or introducing nanomaterials (e.g., nanoparticles, nanotubes, nanosheets) onto the fiber surface to increase surface roughness and specific surface area, as well as to introduce specific functionalities [59]. Particularly, nanomaterial-based modifications employing carbon nanotubes, graphene, SiO_2_, or ZnO have demonstrated effectiveness in enhancing interfacial performance while mitigating fiber buckling under mechanical loads [60]. These modification strategies serve dual purposes: (1) introducing chemically active functional groups to the fiber surface, and (2) increasing surface roughness at micro/nano scales. Through these mechanisms—either by chemical activation or physical topography alteration—significant improvements in AFRP interfacial properties can be achieved. Palola et al. [61] comprehensively reviewed contemporary surface treatment methodologies for aramid fibers and their corresponding effects. The interfacial shear strength (IFSS) of untreated aramid fibers typically ranges from approximately 5–15 MPa. After surface modification (e.g., plasma treatment, chemical grafting), the IFSS can increase to approximately 15–35 MPa.

**Figure 2:**
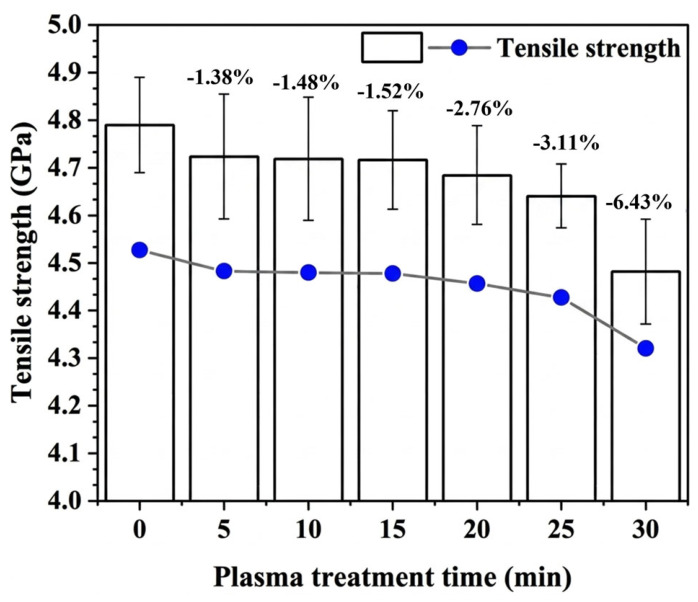
Interfacial performance and tensile properties of aramid fiber after plasma treatment, data from [57], and author redrawn.

However, while treatment methods such as plasma etching can enhance the interfacial bonding performance between aramid fibers and the resin matrix by increasing the fiber surface roughness, they often lead to a degradation of the fiber’s intrinsic mechanical properties, particularly tensile strength [57]. As shown in Figure 2, prolonged bombardment of aramid fiber surfaces with oxygen plasma can alter the molecular chain orientation and integrity of the fiber core layer, leading to internal defects or microcracks within the fibers, thereby reducing the tensile strength of individual filaments. After 30 min of continuous treatment, the tensile strength of the fibers decreased by 6.43% [57]. Furthermore, most existing interfacial modification techniques remain at the laboratory stage, characterized by complex procedures and prolonged treatment durations. Their effectiveness in the context of continuous aramid fiber production lines remains uncertain, raising concerns about their suitability for large-scale civil engineering applications. Additionally, the high cost of certain modification methods may hinder their economic feasibility in civil engineering projects. Another challenge lies in the compatibility of aramid fibers with the diverse resin systems commonly used in civil engineering, as their interfacial adhesion behavior remains poorly understood. Given that weak fiber–matrix interfacial bonding is a major bottleneck limiting the development and application of AFRPs in civil engineering—significantly influencing the overall performance of the composites—future research must prioritize the development of a simple, cost-effective, and industrially scalable interfacial modification method suitable for continuous large-scale production.

## 3. Application of Aramid Fiber and Its Composite in Civil Engineering

### 3.1. Applications in Structural Reinforcement

The application of FRP composites for structural strengthening originated in the 1980s in developed nations such as Japan and the United States. Following decades of global research advancements, the American Concrete Institute (ACI) established the first design guidelines for FRP-strengthened concrete structures with the publication of ACI 440.1R-01 in 2001, which has since undergone continuous refinement through subsequent revisions. FRP-based strengthening techniques are principally classified into three categories: (1) wrap strengthening, (2) externally bonded reinforcement, and (3) near-surface mounted (NSM) reinforcement [62]. Wrap strengthening involves the circumferential confinement of structural members (e.g., beams, columns, and walls) with FRP fabrics or tapes to improve their flexural, shear, and compressive capacities. Externally bonded reinforcement employs FRP laminates or tapes adhesively bonded to structural surfaces, typically applied to repair cracks, enhance flexural performance, or upgrade seismic resistance. AFRPs have garnered significant attention in the field of structural reinforcement due to their high strength-to-weight ratio, corrosion resistance, and excellent energy absorption properties. This section examines their practical applications in structural reinforcement, focusing on performance advantages, limitations, and key implementation challenges.

#### 3.1.1. Confined Concrete Columns

Among the earliest engineering applications of FRP composites was the seismic retrofitting and strengthening of bridge piers and columns. The confinement mechanism of FRP on concrete columns manifests through several key mechanical improvements: (1) enhanced compressive strength, (2) improved ductility, (3) increased confinement effectiveness, (4) greater plastic deformation capacity, and (5) modified failure modes [63]. These confinement effects exhibit significant dependence on multiple parameters, including the compressive strength of the concrete, cross-sectional geometry of the column, and the number of FRP confinement layers.

**Figure 3:**
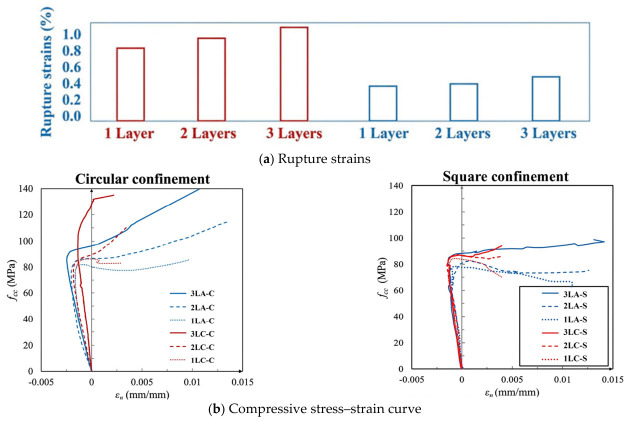
Comparison of compressive properties between AFRP- and CFRP-confined concrete columns, data from [65,66], and author redrawn.

Aramid fibers are employed to confine and reinforce compressive concrete columns, enhancing their axial load-bearing capacity and resistance to lateral deformation. This method also improves the ductility and toughness of the columns [64]. Specifically, AFRP wrapping effectively restricts the lateral expansion of concrete and improves seismic performance. In comparison to carbon and glass fibers, aramid fibers contribute to both strength and ductility enhancements. In comparison to carbon and glass fibers, aramid fibers contribute to both strength, ultimate rupture strain, and ductility enhancements under both circular and square confinements, as shown in Figure 3, Table 2f,j,o [65,66]. G. Wu et al. [67] investigated the axial compression behavior of concrete columns confined with carbon, glass, and aramid fiber sheets. They observed that high-strength, high-modulus CFRP enhances confinement pressure, significantly improving the strength of concrete columns and effectively restraining concrete expansion under high stress levels, thereby increasing both strength and stability. However, CFRP is prone to brittle fracture once its stress threshold is reached, resulting in limited ductility improvement [68]. In contrast, although GFRPs and AFRPs possess lower elastic module and provide less pronounced confinement than CFRPs, they can sustain increasing confinement effects after the concrete reaches its peak strength, mitigating brittle failure and improving ductility. Compared to CFRPs, GFRPs exhibit higher ductility but offer less strength enhancement. AFRPs demonstrate intermediate effectiveness between CFRPs and GFRPs in improving both the strength and ductility of concrete. Jian C. Lim et al. [69] also compared the influence of AFRPs, CFRPs, GFRPs, and active confinement on the axial compressive behavior of concrete. Their results indicate that CFRP generally provides the highest strength enhancement but with low ductility, whereas GFRPs offer good ductility with relatively lower strength improvement, as shown in Table 2e. AFRPs exhibit a more balanced performance between strength and ductility. Additionally, some researchers have explored hybrid confinement using different fiber materials (e.g., combining high-strength CFRPs with high-ductility GFRPs or AFRPs) to synergistically enhance both the strength and ductility [70]. However, if the material properties differ too significantly or the proportion of high-strength to low-strength materials is inappropriate, the hybrid effect may be suboptimal, potentially preventing each material from contributing effectively.

The axial compressive behavior of AFRP-confined concrete columns is influenced by factors such as concrete strength, number of AFRP layers, as well as the cross-sectional shape and size of the column. Han-Liang Wu et al. [72] investigated the factors affecting the performance of high-strength concrete (HSC) columns confined with AFRP under axial compression, as show in Table 2b. Their findings indicate that: (1) Due to the inherent density and high compressive resistance of high-strength concrete, its lateral deformation under confinement is relatively small. As a result, the confining effect of AFRP is more pronounced in low-strength concrete, where it acts primarily as a strengthening mechanism; (2) Increasing the number of AFRP layers enhances the confinement effect, leading to significant improvements in both compressive strength and ductility. However, the rate of improvement diminishes beyond a certain number of layers; (3) Continuous AFRP wrapping considerably improves both the strength and ductility of columns, whereas discontinuous wrapping enhances strength but has limited effect on ductility. Yuan-Feng Wang et al. [80] studied the size effect in AFRP-confined short concrete columns. Their research revealed that while the size effect has minimal influence on the failure mode and stress–strain curve, it significantly affects strength. Specifically, under low AFRP confinement ratios, smaller specimens demonstrated greater strength enhancement. At higher confinement ratios, however, the size effect became less noticeable or even negligible.

**Figure 4:**
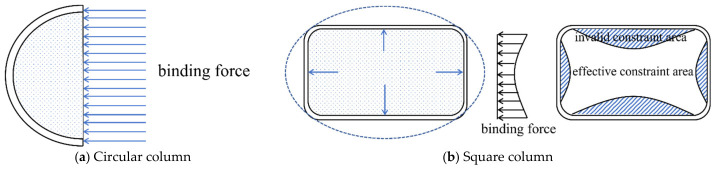
Effective confined area of circular column and square column.

**Figure 5:**
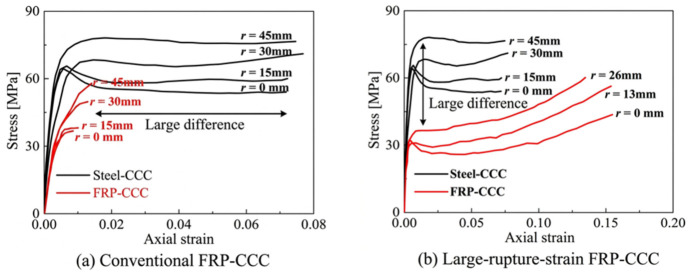
Comparison of compressive stress–strain curves between conventional FRP and large rupture strain FRP, data from [70], and author redrawn.

The effectiveness of FRP confinement is also strongly affected by the cross-sectional shape of the column. As illustrated in Figure 4, in circular columns, the FRP jacket conforms tightly to the surface, ensuring uniform force distribution and providing effective confinement to the core concrete. This results in significant improvements in both load-bearing capacity and ductility. In contrast, for rectangular columns, the FRP jacket does not fit snugly, leading to bending deformation along the flat sides [70]. Given the low flexural stiffness of FRP materials, the confinement pressure exerted on the core concrete in these regions is minimal. Given the low flexural stiffness and limited deformation capacity of conventional FRP materials, the jacket tends to bulge outward along the flat sides under lateral expansion of the concrete, resulting in reduced confinement pressure in these regions. This behavior is closely related to the linear-elastic brittle response and relatively low rupture strain of conventional FRP composites, as illustrated in Figure 5a, which limits their ability to accommodate transverse deformation. As shown in Figure 5b, large-rupture-strain FRP composites are capable of sustaining significantly larger deformation, thereby improving the confinement effectiveness and ductility of FRP-confined concrete columns (FRP-CCC). Therefore, the confinement efficiency in square columns is governed not only by geometric compatibility but also by the deformation capacity of the confining material. Consequently, the corners experience strong confinement due to the perpendicular action of the FRP, creating distinct effective and ineffective confinement zones across the cross-section [81]. The size of the chamfer at the corners of rectangular columns plays a critical role in the confinement efficiency [82]. An excessively small chamfer radius intensifies stress concentration in the FRP at the corners, reducing its overall confining effect and limiting the enhancement in bearing capacity and ductility [83].

**Figure 6:**
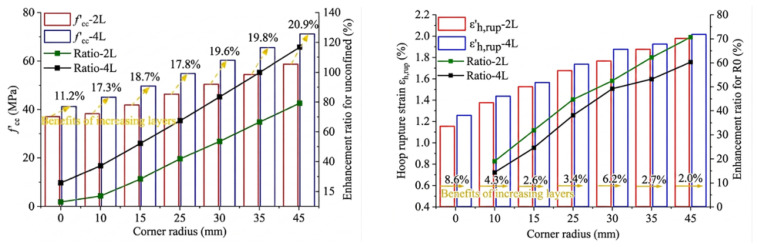
Effect of corner radius on ultimate strength and hoop rupture strain, data from [82], and author redrawn.

Figure 6 presents a comparative analysis of the confinement effects provided by FRP jackets on square concrete columns with varying levels of ductility. As discussed in the manuscript (Figure 6), the compressive strength, axial strain, and hoop strain all increase with the number of BFRP layers. However, the marginal benefit decreases with increasing thickness. For example, when the corner radius was 0 mm, 25 mm, and 45 mm, two-layer confinement increased the compressive strength by 13.2%, 41.9%, and 79.0%, respectively, compared to unconfined specimens, while the additional gain from increasing to four layers was only 11.2%, 17.8%, and 20.9%. A similar trend was observed for hoop strain, where the maximum improvement from additional layers was limited (approximately 8.6%). Both CFRPs and GFRPs exhibited relatively poor shear performance. Their limited rotation capacity at small chamfers prevented full conformity to the profile, resulting in large ineffective confinement areas. In comparison, aramid fiber demonstrated superior shear resistance and greater allowable rotation angle, enabling better conformity at chamfered corners. The resulting ineffective confinement area was smaller than that of the CFRP or GFRP wraps. Therefore, AFRPs are expected to provide more effective confinement for square or rectangular concrete columns. However, the relationship between corner radius and confinement performance is influenced by multiple interacting factors, including section size, FRP type, thickness, and material properties. Therefore, a unified design limit for the critical corner radius-to-thickness ratio (r/t) has not yet been established in the literature. Comparing the compressive stress–strain curves between conventional FRP and large rupture strain FRP, the latter showed more ultimate compressive strength and axial deformation, as shown in Figure 6. Therefore, as a large rupture strain FRP, AFRP is expected to provide more effective restraint effects for square or rectangular concrete columns [70].

Conventional reinforced concrete (RC) columns are susceptible to premature failure under seismic loading due to insufficient ductility, brittle fracture mechanisms, or high slenderness ratios. The application of AFRP confinement has been shown to substantially enhance the seismic performance of such columns. Mohsen et al. [84] conducted a comparative study on AFRP-confined and GFRP-confined RC columns. Their findings indicate that active AFRP confinement with prestressing levels of 10% and 30% both contributed to improved seismic behavior. The higher prestressing level (30%) resulted in superior performance compared to the 10% condition, exhibiting greater enhancements in strength, ductility, energy dissipation capacity, and damage control. Moreover, the improvement observed in AFRP-confined RC columns was more significant than that in their GFRP-confined counterparts. To et al. [83] investigated the behavior of AFRP-strengthened RC columns under seismic conditions. Their study demonstrated that the application of AFRPs resulted in substantial improvements in both strength and ductility. In comparison to unreinforced specimens, the AFRP-confined columns exhibited superior seismic resistance, characterized by delayed stiffness and strength degradation, as well as enhanced energy dissipation capacity. Similarly, Henni et al. [85] examined the performance of RC columns strengthened with AFRP sheets under cyclic loading. The results indicated a notable increase in ductility, with the compressive strength rising from 23.1 MPa for unconfined concrete to 53.7 MPa after confinement. Furthermore, the energy dissipation capacity was enhanced by a factor ranging from 1.96 to 3.29. An optimal AFRP bond length was identified to be between 100 mm and 150 mm. Compared to CFRPs, AFRPs provide higher ductility, which allows concrete columns to undergo larger deformations under seismic actions. This increased deformation capacity enables greater energy absorption, reducing the likelihood of brittle failure under repeated earthquake loading.

Additionally, AFRPs offer superior corrosion resistance and tolerance to high temperatures, contributing to the improved durability and fire performance of confined concrete members. Talikoti et al. [86] conducted experiments on concrete cube specimens wrapped with double-layer AFRP and subjected to aggressive environments. When immersed in a dilute hydrochloric acid solution for curing periods of 7, 30, and 70 days, the AFRP-confined specimens exhibited a 40% reduction in weight loss and a 140% increase in compressive strength relative to the unconfined specimens. In fire resistance tests conducted at 200 °C for varying durations, the confined samples showed a 60% reduction in weight loss and approximately 150% higher compressive strength retention, confirming the beneficial effect of AFRPs in harsh conditions.

In summary, the confinement effect provided by AFRPs on concrete columns is intermediate between that of CFRPs and GFRPs, offering a balanced overall performance. This is particularly advantageous for square cross-section columns, where AFRPs demonstrate notable effectiveness. Additionally, due to its high toughness and superior corrosion resistance, AFRP-confined concrete columns exhibit enhanced seismic performance and durability under high-temperature conditions. The efficiency of AFRP confinement is highly sensitive to the interfacial bond behavior between aramid fiber layers and the matrix. However, several challenges remain: first, the inherent poor adhesion between aramid fibers and resin matrices often leads to interfacial debonding, preventing the full utilization of the fiber’s mechanical properties. Second, the effective overlap length for AFRP sheets has not been clearly established. Although existing studies often maximize the lap length in practice, specific design values are seldom provided. Third, as a consequence of the aforementioned issues, the performance improvement of square columns confined with AFRP was not as pronounced as that observed in circular columns. Nevertheless, given the inherent toughness of aramid fibers, AFRPs remain a promising solution for enhancing the confinement of square columns. With improvements in the bonding performance between aramid fibers and resin matrices, AFRP-confined concrete columns are expected to offer significant application potential in future structural engineering practice.

#### 3.1.2. Strengthening Concrete Beams

AFRP composites have emerged as an important strengthening solution for enhancing the structural performance of concrete beams owing to their advantageous properties such as high strength, low weight, corrosion resistance, and ease of installation. Recent research has increasingly focused on the application of AFRPs in improving critical mechanical behaviors of beams, including flexural, shear, and torsional resistance, with substantial progress having been made in these areas over the past years.

The effectiveness of FRP composites in the flexural strengthening of concrete beams is influenced by several factors, including the strengthening configuration, type of FRP material, as well as the thickness and number of composite layers. Common strengthening configurations include bonding FRP to the soffit (bottom gluing), U-jacketing, full wrapping, and side bonding. Among the commonly used FRP types, such as CFRPs, GFRPs, and AFRPs, CFRPs provide the most substantial improvement in the flexural capacity of beams. This is attributed to its high elastic modulus and tensile strength, which enable it to effectively share the tensile stresses at the beam’s bottom. In contrast, GFRPs yield the least improvement [87]. Sing et al. [88] demonstrated that the use of one to three layers of GFRP can enhance the ultimate bearing capacity of beams by 18% to 46%. Under the bottom bonding configuration, Michael et al. [89] reported that the strengthening effectiveness of AFRPs lies between that of CFRPs and GFRPs; however, AFRP-reinforced beams exhibited the most ductile behavior. Their study also indicated that AFRP sheets significantly improve both static and fatigue performance. In a comparison of the flexural behavior of unbonded prestressed beams strengthened with AFRPs and CFRPs, He et al. [90] found that the stress distribution and ultimate bearing capacity were nearly identical for the two materials at the same number of layers, with the difference being within 3%. Zhang and Wang [91,92] demonstrated that AFRP sheets can effectively enhance the flexural capacity and stiffness of beams under static loading. For instance, applying three layers of AFRP was shown to increase the ultimate load by up to 91% and reduce mid-span deflection by 50%. However, the strengthening efficiency exhibits a nonlinear decreasing trend with increasing fiber dosage: a significant improvement is observed initially, which slows down in later stages due to diminishing returns. However, excessive AFRP application may lead to a change in failure mode from ductile flexural failure to premature interface debonding. This transition is mainly attributed to the increased stiffness of the AFRP reinforcement, which induces higher interfacial stresses and strain incompatibility between the AFRP and the concrete substrate, thereby suppressing the yielding of internal steel reinforcement and reducing the overall ductility. Under fatigue loading, AFRP sheets effectively restrain crack development, resulting in an increased number of cracks, reduced crack spacing, smaller crack widths, and limited crack propagation, which collectively enhance the beam’s fatigue crack resistance. Nevertheless, over-reinforcement (e.g., two layers compared to one layer) may lead to a reduction in fatigue life, the additional interface introduces stress concentrations under cyclic loading, leading to premature interfacial shear failure, as reported by [92] with a decrease of up to 35%, that is, the reduction in fatigue life for the two-layer AFRP-strengthened beam is predominantly explained by intermediate surface damage (interlaminar debonding), with microcrack development playing a secondary role. Jamshid Ruziev et al. [93] investigated the flexural performance of recycled coarse aggregate concrete beams strengthened with para-aramid fiber sheets, comparing the effectiveness of aramid mesh with different spacings and bonding methods (bottom bonding vs. U-jacketing). Their findings indicated that U-jacketing with two layers increased the flexural strength by up to 24%, while a mesh spacing of 14 mm provided the best ductility. The study concluded that aramid fiber sheets can effectively compensate for the performance loss induced by recycled aggregates, offering a viable solution for sustainable construction. Furthermore, AFRPs are also applicable for rehabilitating damaged beams. Rameshkumar U More et al. [94] examined the influence of externally bonded AFRPs on the flexural behavior of reinforced concrete beams with varying damage levels (0%, 70%, 80%, 90%, and 100%). The results revealed that for undamaged beams, single-layer and double-layer AFRP reinforcement increased the load-bearing capacity by 27.59% and 48.27%, respectively. Beams with 70% and 80% damage also exhibited significant recovery in performance after strengthening. AFRP reinforcement led to reduced deflection at ultimate load, increased stiffness, fewer cracks, smaller crack widths, and slower crack propagation. Similarly, Deng et al. [95] confirmed that AFRP sheets can substantially restore the flexural performance of corroded beams. For moderately corroded beams (with a steel mass loss rate of 6.0%), the ultimate load and deflection were increased by 83% and 166%, respectively.

In terms of shear strengthening, a variety of AFRP composites have demonstrated considerable potential. Adhikary et al. [96] compared the shear performance of concrete beams strengthened with CFRPs and AFRPs in a U-wrapped configuration. Their results indicated that AFRP reinforcement could enhance the shear capacity by up to 118%, a value comparable to that achieved with CFRPs (123%). Similarly, Ruben et al. [97] reported that double-layer AFRP wrapping increased the ultimate shear capacity of both solid and hollow beams by 36%, with the double-layer configuration proving significantly more effective than a single layer (which provided a 14% increase). A more innovative approach involves the use of continuous aramid fiber ropes for shear strengthening, leveraging advantages such as low weight, high strength, durability, and ease of installation. Sekijima et al. [98] experimentally investigated the use of aramid fiber ropes in the shear span region of reinforced concrete (RC) beams, including those with pre-induced shear damage. Their findings showed that applying aramid ropes could alter the failure mode of beams without shear reinforcement from shear failure to flexural-tension failure, increase the ultimate load-carrying capacity by more than twofold, and effectively restore the performance of shear-damaged beams. Furthermore, Soo and Hong [99] employed aramid fiber prestressed strips (AFPS) for combined flexural and shear strengthening. This method increased the load resistance of flexural failure specimens by 164% to 193%, and led to an even more pronounced improvement (218% to 259%) in specimens that failed in shear.

Torsional failure, a common brittle failure mode in RC beams subjected to dynamic loads such as earthquakes, is characterized by rapidly propagating diagonal cracks that lead to sudden structural collapse. This failure mode poses a significant safety risk, particularly in older structures where torsional resistance was not fully considered in the original design. To enhance the torsional performance of existing structures, FRP composites have emerged as an efficient and economical strengthening method. Among these, AFRPs are particularly advantageous due to its high tensile strength and deformation capacity, enabling it to withstand principal tensile stresses under torsion and delay concrete cracking. Furthermore, its high toughness allows the concrete to continue carrying load after cracking, thereby mitigating brittle failure mechanisms. S. Shanmugasundaram et al. [100] compared the effectiveness of CFRPs and AFRPs in enhancing the torsional behavior of concrete beams under different wrapping schemes. The results demonstrated that AFRPs provided a higher torsional capacity compared to CFRPs. Beams strengthened with AFRPs also exhibited greater deformation capacity owing to AFRPs’ superior toughness and ductility, whereas CFRP-strengthened beams were prone to abrupt fracture. The torsional capacity of AFRP-strengthened beams was 40% to 140% higher than that of the unstrengthened beams, depending on the wrapping angle. Specifically, AFRPs with 90° wrapping showed a 6.91% higher capacity than CFRPs with the same configuration, while at a 45° angle, the advantage of AFRPs increased to 16%. In a related study, Sachin B. Kandekar et al. [101] investigated the torsional behavior of M30-grade RC beams fully wrapped with aramid fiber sheets. Their findings indicated that AFRP wrapping increased the cracking moment and ultimate torsional moment by 140%. Additionally, Kandekar et al. [102] observed that using AFRP strips with closer spacing resulted in more effective lateral confinement, which effectively restrained crack development. In contrast, larger strip spacing left unreinforced sections where cracks could propagate, thereby reducing the strengthening efficiency. For instance, beams strengthened with 100 mm wide AFRP strips spaced at a certain interval exhibited an 80% increase in cracking and ultimate torsional moments [102]. Further research by the same author [103] on pre-damaged beams showed that fully wrapped AFRP restoration recovered 105% of the original torsional capacity, while strip wrapping restored 92%. CFRPs, with their low ultimate strain and brittle failure mode, tend to fracture abruptly once the cracking strain is exceeded, leading to sudden loss of confinement and torsional capacity. In contrast, AFRPs exhibit a much higher ultimate strain and progressive failure behavior. AFRPs significantly improve the torsional performance of concrete beams, attributable to its high toughness and ductility. It is important to note, however, that the effectiveness of AFRP strengthening is highly dependent on the bond behavior at the AFRP–concrete interface. Superior interfacial bonding ensures effective stress transfer between the AFRP and the concrete substrate during torsion, delays debonding failure, and thereby enhances the overall torsional performance of the strengthened member.

In summary, AFRP composites—including sheets, plates, ropes, meshes, and strips—have demonstrated significant effectiveness in enhancing the flexural, shear, and torsional performance of concrete beams. While the improvement in flexural capacity typically falls between that provided by CFRPs and GFRPs, AFRP strengthening often results in superior structural ductility. The effectiveness of AFRP reinforcement is influenced by multiple factors, such as the fiber dosage, bonding technique, substrate condition (e.g., degree of corrosion), type of loading (static or fatigue), and the specific strengthening configuration. Consequently, the selection of an appropriate reinforcement strategy should be tailored to the specific engineering scenario. A critical aspect governing the performance of AFRP-strengthened beams, as with other FRP systems, is the bond behavior at the concrete–composite interface. The effectiveness of aramid fiber-reinforced polymer (AFRP) strengthening systems is fundamentally governed by the interfacial bonding behavior between the AFRP and concrete substrates. The load transfer across the AFRP–concrete interface is primarily achieved through a combination of mechanical interlocking, chemical adhesion, and frictional resistance. However, compared with carbon fibers, aramid fibers possess relatively smooth and chemically inert surfaces, which limits their interfacial adhesion and makes the fiber–matrix interface a potential weak link in the stress transfer path. Consequently, the bond performance of AFRP-strengthened systems is highly sensitive to factors such as fiber surface treatment, adhesive properties, concrete surface preparation, and environmental conditions including moisture and temperature variations. Typical failure modes include interfacial debonding, cohesive failure within the concrete substrate, and fiber pull-out, with AFRP systems exhibiting a higher tendency toward interfacial-related failures. This premature failure mode frequently prevents the full utilization of the FRP’s high strength. Therefore, future research should continue to prioritize the investigation of bond performance, as it remains central to optimizing the effectiveness of AFRP strengthening. Building upon a sound understanding of interfacial behavior, further systematic studies are needed to quantify the influence of key parameters—such as the number of layers and total thickness of AFRP—on the ultimate capacity, ductility, and crack control of strengthened beams. Establishing quantitative relationships for these parameters is essential for refining design guidelines and ensuring reliable application.

#### 3.1.3. AFRPs for Blast-Resistant Concrete Structures

In light of the growing incidence of terrorist attacks, bombings, and other impact events, enhancing the impact resistance of existing RC structures has become a critical priority. Traditional strengthening techniques, such as bonding steel plates or enlarging cross-sections, suffer from limitations including complex construction processes, significant increases in self-weight, and susceptibility to corrosion. Moreover, under impact or blast loading, the fragmentation of RC members can lead to severe secondary disasters. When subjected to impact, penetration, or explosive loads, concrete slabs typically exhibit brittle failure modes—characterized by localized crushing and radial cracking on the impacted side, as well as concrete spalling and scabbing on the rear side, which produces high-velocity fragments and exacerbates collateral damage. FRP composites have been widely adopted for the flexural and shear strengthening of RC structures due to their high strength-to-weight ratio, corrosion resistance, and ease of installation. Among them, AFRPs exhibit exceptional impact resistance, attributable to their high elongation at break and superior toughness compared to CFRPs. Under dynamic loading, AFRPs can absorb and dissipate substantial strain energy through mechanisms such as fiber stretching, fracture, and interfacial debonding. Among them, AFRPs exhibit better impact resistance, attributable to their high elongation at break and superior toughness compared to CFRPs and GFRPs. Under dynamic loading, AFRPs can absorb and dissipate more strain energy through mechanisms such as fiber stretching, fracture, and interfacial debonding. These properties make them suitable for personal ballistic protection—as in bulletproof helmets and vests—and also highly effective in structural strengthening applications. When used to retrofit concrete slabs, AFRPs can significantly improve structural integrity, toughness, and overall damage resistance under extreme dynamic conditions such as impact, explosion, and penetration. The strengthening mechanisms include restraining crack propagation, preventing rear-face spalling, providing membrane tension capacity, and enabling efficient energy dissipation through the aforementioned deformation processes.

The impact resistance of AFRP-strengthened concrete columns has been extensively investigated under various loading conditions. Under axial impact, the confinement mechanism of the AFRP significantly enhances dynamic performance. Hui Yang et al. [104] studied the compressive properties of AFRP-confined concrete at high strain rates (80–170 s^−1^), finding that the dynamic compressive strength, ultimate strain, and energy absorption capacity were markedly superior to those of unconfined concrete. Further research by Hengwen Song et al. [105] on the dynamic behavior of AFRP-wrapped circular concrete specimens under repeated impacts revealed that the AFRP confinement effectively maintained structural integrity and load-bearing capacity, even under repeated high-strain-rate impacts (30–60 s^−1^). The peak stress remained stable (with fluctuations of less than 15%), and impact toughness showed no significant attenuation. This performance is attributed to the lateral confinement provided by AFRP, which places the concrete in a triaxial stress state and alters the energy absorption mechanism. Although internal damage in the concrete accumulates linearly with the number of impacts, most of the impact energy is stored and dissipated as releasable strain energy through the elastic deformation of the AFRP. Comparative studies have further elucidated the role of fiber properties. Yan et al. [106] compared the axial impact behavior of concrete cylinders confined with aramid fiber (~3% rupture strain) and large-rupture-strain (LRS) FRP (~8% rupture strain). Their results indicated that while the AFRP, with its high modulus and strength, effectively increased the peak impact force, the LRS FRP with similar circumferential stiffness but greater fracture strain demonstrated superior energy absorption. This was reflected in a significantly extended impact duration, a distinct energy absorption plateau in the force–time history curve, and more effective mitigation of damage to the core concrete. The study also confirmed that increasing the number of FRP layers generally enhances impact resistance. Under lateral impact and blast loading, AFRPs also demonstrate effective strengthening. Gurbuz et al. [107] conducted full-scale drop-hammer tests on square reinforced concrete columns, finding that unreinforced specimens failed in a brittle shear-bending mode, whereas those wrapped with two layers of AFRP fabric exhibited significantly improved impact resistance, withstanding higher impact energy and failing in a more ductile bending mode characterized only by vertical cracks and reduced residual deformation. The AFRP retrofit effectively inhibited concrete spalling, controlled crack development, and enhanced the shear strength of the members, thereby improving the overall dynamic response. A systematic numerical study by Hosseini et al. [108] on the effect of blast loading on CFRP- and AFRP-strengthened RC columns further demonstrated that at similar thicknesses, the AFRP-strengthened columns exhibited superior blast resistance compared to their CFRP counterparts. This advantage fundamentally stems from the inherent high toughness and ductility of AFRPs, which allows them to absorb and dissipate more energy through greater plastic deformation, fiber pull-out, and interfacial debonding, whereas the brittle nature of CFRPs often leads to sudden fiber fracture.

In the context of strengthening concrete beams against impact loading, existing research demonstrates that externally bonded FRP sheets can significantly enhance impact resistance and reduce maximum deflection. A critical failure mode observed under such conditions is FRP debonding, where the dynamic interfacial bond behavior between FRP and concrete exhibits a notable strain rate effect, resulting in a lower debonding strain compared to quasi-static conditions [109]. Experimental comparisons by Taiping Tang et al. [110] between carbon and aramid fiber reinforcement revealed that while both improved the impact resistance, the aramid-strengthened beams exhibited superior deflection tolerance and a more gradual failure process. These beams developed multiple cracks and underwent noticeable deformation prior to failure, absorbing more energy under repeated impacts and demonstrating better toughness. In contrast, the carbon fiber-reinforced beams failed more abruptly with fewer cracks. Le Huy Sinh et al. [111] further quantified the effectiveness of AFRPs, reporting reductions in maximum and residual displacement under impact by 35% and 85%, respectively. It is noteworthy that impact loading typically alters the failure mode of beams from flexure-dominated under static loads to shear–flexure interaction. Under low-energy impacts, concrete crushing tends to precede AFRP fracture, whereas high-energy impacts lead to significant FRP debonding. The study by Kurihashi et al. [112] corroborates the excellent recovery performance of AFRP-plated beams after impact, attributable to the high elasticity and strength of aramid materials. In summary, under impact loads, the failure of RC beams shifts from flexural to shear-influenced mechanisms, with FRP debonding representing a key challenge. This is compounded by the strain-rate sensitivity of the FRP–concrete interface, which leads to a reduction in dynamic debonding strain.

Research on strengthening concrete slabs with aramid fibers demonstrates their effectiveness under extreme loads. Zhen Gao et al. [113] evaluated the penetration resistance of slabs with aramid fibers bonded to either the impact face or the rear face. Their results indicated that rear-face bonding offered superior performance. While front-side bonding increased the penetration resistance by 10.41% compared to an unstrengthened slab, the rear-bonded aramid layer was particularly effective in suppressing back-face spalling and reducing the tensile damage by attenuating stress waves throughout the penetration process. In a subsequent study, Zhen Gao et al. [114] further demonstrated that bonding aramid laminates to the rear face could significantly enhance the blast resistance against 45 g and 75 g TNT charges. This configuration prevented concrete spalling and helped maintain structural integrity. The study identified an optimal thickness ratio of 50:3 (concrete to AFRP) for a 50 mm thick slab. Kong et al. [115] numerically confirmed these benefits by developing a refined finite element model that accounted for high strain rate effects and dynamic interfacial behavior. Their simulation results showed that AFRP strengthening changed the failure mode from concrete spalling and flexural failure to AFRP fracture and debonding. This shift effectively delayed concrete cracking, reduced radial cracks, and inhibited spalling, leading to a 50.9% reduction in rebar stress and a 24.3% decrease in maximum displacement.

In summary, AFRPs can effectively enhance the resistance and toughness of concrete structures under impact and blast loading, with high-toughness aramid fibers demonstrating significant application potential. Current research indicates that no definitive conclusion has been reached regarding the comparative effectiveness of carbon fibers and aramid fibers in improving the impact resistance of concrete structures, as their performance is influenced by multiple factors such as fiber content, impact velocity, impact location, and angle. Although the failure modes of AFRP-strengthened members vary under impact loading at different velocities, their failure behavior remains primarily controlled by the bond performance between the AFRP and concrete. Similar to quasi-static strengthening, the interfacial bond behavior remains a key factor in evaluating and optimizing the strengthening effect in future studies. It is worth noting that due to the significant strain rate effect of this bond performance, the failure mechanisms of AFRP-strengthened structures under dynamic loading also change accordingly. Therefore, establishing a reliable predictive model for the impact resistance of AFRP-strengthened concrete structures must first clarify the fracture energy parameters of the AFRP–concrete interface under different strain rates. In this context, developing test and characterization methods for the bond fracture energy between AFRP and concrete applicable under different strain rate conditions has become a critical issue that needs to be urgently addressed in this field.

### 3.2. AFRP Bars/Rods

#### 3.2.1. AFRP Bar-Reinforced Concrete

Traditional steel reinforcement is susceptible to corrosion, particularly in harsh environments, which substantially compromises the durability and service life of structures. Although UV and a moisture environment might lead to the corrosion and degradation of aramid fiber, under the protection of the AFRP inner matrix and outer concrete, AFRP bars have significantly higher strength, corrosion resistance, and low density than traditional steel. Thus, AFRP bars have shown considerable potential in civil engineering. As internal reinforcement for concrete beams, AFRP bars have shown considerable potential in civil engineering due to their high tensile strength, corrosion resistance, and low density. However, a significant drawback is their substantially lower elastic modulus—approximately 30% less than that of conventional steel reinforcement. This characteristic can lead to a reduction in structural stiffness, increased deflection under load, and a tendency toward brittle post-cracking behavior. Consequently, this limitation represents a key obstacle to the broader application of AFRP bars.

AFRP bars are primarily composed of highly oriented aramid fibers (such as Twaron^®^ and Nomex^®^) embedded in an epoxy or vinyl ester resin matrix, typically with a fiber volume fraction ranging from 45% to 65%. These composites offer advantages including low flammability, high-temperature resistance (with a degradation temperature around 500 °C), non-magnetic properties, and resistance to organic solvents. However, their performance is comparatively weaker in terms of acid resistance, salt corrosion resistance, and UV resistance. The typical diameter of AFRP bars ranges from 4 to 20 mm. They exhibit a tensile strength of 1200–2324 MPa (approximately 1.0–1.2 times that of equivalent-grade steel), an elastic modulus of 50–131 GPa (about 25–65% of steel’s modulus), and a density of only 1250–1400 kg/m^3^, which is significantly lower than that of steel. In existing research, Rafieizonooz et al. [116] provided a systematic summary of the types and performance characteristics of AFRP bars. Separately, Medina [117] conducted tensile, creep rupture, and stress relaxation tests on Arapree^®^ AFRP bars, reporting a tensile strength of approximately 1460 MPa, an elastic modulus of 70.6 GPa, and a failure strain of 2.1%. The results indicated typical linear elastic behavior, and it was noted that the stress relaxation loss is closely related to the initial stress level and environmental conditions.

The bond performance between AFRP bars and concrete is crucial for ensuring their effective collaboration in structural systems and the reliable transfer of internal forces. In comparison, the bond strength of AFRP bars is approximately 85% of that of GFRP or CFRP bars and is influenced by various factors, such as bar surface morphology, concrete properties, and environmental conditions [118,119]. Thus, clarifying the bond-slip constitutive model between AFRP bars and concrete, along with further enhancing their interfacial performance, is key to advancing the practical application of AFRP bars in concrete structures.

In the study of AFRP-reinforced concrete beams, there has been considerable research on their flexural performance, yet investigations into their shear and torsional behavior remain limited. Through four-point bending tests, M. A. Rashid et al. [120] found that high-strength concrete beams reinforced with AFRP bars exhibited greater flexibility after cracking compared to equivalent steel-reinforced beams, with predominantly vertical and wider cracks. This also highlighted issues such as insufficient ductility, poor deformation capacity, and excessive crack widths in purely AFRP-reinforced concrete beams. Therefore, hybrid reinforcement has emerged as an effective approach to balancing load-carrying capacity and ductility. High-strength concrete beams with a combination of AFRP bars (in tension) and steel bars (in shear/compression) display a stiffness comparable to that of steel-reinforced beams before cracking. However, after cracking, stiffness drops sharply due to the low elastic modulus of AFRP, resulting in rapid crack propagation. Under specific loads, both crack width and deflection increase significantly [120]. Further research indicates that increasing the AFRP reinforcement ratio in hybrid systems can enhance the cracking load and ultimate load while improving the crack distribution; however, excessive AFRP reinforcement leads to a substantial increase in deflection [121]. R. A. Hawileh [122] developed a nonlinear finite element model capable of accurately simulating steel–AFRP hybrid reinforced concrete beams, demonstrating that this reinforcement method significantly improves the load-bearing capacity while maintaining ductility. Moreover, similar to CFRP, AFRP reinforcement has also been applied in prestressed structural systems.

In prestressed structures, the bond performance of AFRP reinforcement is particularly critical. Studies have shown that prestressed AFRP-reinforced beams behave similarly to prestressed steel-reinforced concrete beams in the linear elastic stage prior to cracking. However, after cracking, their deflection increases more significantly due to the lower elastic modulus of AFRPs while maintaining an approximately linear response up to failure [123]. To mitigate the brittle failure mode associated with AFRPs, innovative bond-state strategies—such as the use of unbonded reinforcement, a hybrid configuration of bonded and unbonded tendons, or the addition of non-prestressed reinforcement—can significantly enhance the ductility, reduce crack density, and improve toughness. Nevertheless, it should be noted that bonded reinforcement contributes more substantially to the ultimate load-carrying capacity [124]. Further studies by Saafi [125] and Kim et al. [126] demonstrated that prestressed concrete beams employing bonded–unbonded hybrid reinforcement or supplementary non-prestressed AFRP reinforcement can achieve an ultimate deflection up to 2.7 times greater than conventional designs. The ductility index increased from 1.8 to 4.5, and the failure mode shifted from brittle reinforcement rupture to a gradual, multi-crack slip mechanism, thereby effectively alleviating the brittle fracture tendency typical of FRP reinforcement.

Prestress relaxation represents a key issue that must be clarified before AFRP reinforcement can be applied to prestressed structures. Numerical studies conducted by Lou et al. [127] indicate that if the stress relaxation of AFRP reinforcement is neglected, the prestress loss due to its low elastic modulus is smaller than that of steel strands, leading to greater camber and smaller load-induced deflection. However, when the significant inherent relaxation of AFRP is considered, the total prestress loss exceeds that of steel strands, resulting in reduced long-term camber and increased load deflection. To address this issue, Youakim and Karbhari [128] proposed an analytical method based on the principle of equilibrium and compatibility, which introduces a relaxation correction coefficient (recommended as 0.95) specifically to account for the relaxation effect of AFRP reinforcement. This method can effectively predict long-term prestress loss, stress redistribution, and deflection variation in such systems.

Introducing aramid fiber-reinforced polymer (AFRP) and steel reinforcement into concrete structures is an effective approach for achieving a balanced combination of mechanical properties. This hybrid system enables complementary performance by incorporating AFRP bars and conventional steel reinforcement within the same member: steel reinforcement provides a high elastic modulus and pronounced yield ductility, whereas AFRP bars offer high specific strength, excellent fatigue resistance, and superior corrosion resistance. Under loading, hybrid-reinforced members typically exhibit distinct staged response characteristics. In the initial stage, both steel reinforcement and AFRP contribute to the overall stiffness; as the load increases, the steel reinforcement gradually yields, while AFRP remains in a linear elastic state and carries additional incremental load, thereby effectively delaying stiffness degradation. At the ultimate limit state, AFRP governs the load-carrying capacity and significantly enhances the ultimate strength of the member. This synergistic mechanism allows the hybrid system to overcome the brittle failure behavior associated with solely AFRP-reinforced members, as steel yielding provides deformation capacity while the AFRP ensures sustained load resistance, resulting in a pseudo-ductile response [122]. In addition, steel reinforcement effectively controls crack initiation and propagation, while the AFRP helps restrain crack growth, thereby improving the serviceability and stiffness of the member. Nevertheless, several challenges remain, including the lack of comprehensive design methodologies, inconsistencies in ductility evaluation criteria, and the complexity of interfacial interaction mechanisms. In particular, the effects of the steel-to-AFRP reinforcement ratio, differences in elastic modulus, and bond performance on the overall structural behavior require further systematic investigation.

Based on manufacturing processes, AFRP bars can be primarily categorized into pultruded and braided types. Pultruded AFRP bars are typically manufactured from continuous long fibers, resulting in a unidirectional composite with relatively higher stiffness. In contrast, braided AFRP bars are produced by weaving resin-impregnated aramid fibers into a rope-like structure. Their stiffness can be adjusted by varying the braiding pitch and the resin matrix, which allows for the production of flexible AFRP bars that can be coiled for transportation and are suitable for externally prestressed structures with large curvatures. Xue et al. [129] investigated the application of flexible AFRP bars in reinforced concrete structures using ARA-type AFRP bars produced by the FiBRA Corporation of Japan. The material properties of these bars exhibit typical linear elastic and brittle behavior, characterized by high ultimate tensile strength (1120–1775 MPa) but a relatively low elastic modulus (49–90 GPa). Additionally, their Poisson’s ratio (approximately 0.6) is significantly higher than that of steel. These core mechanical properties fundamentally account for the structural behavior differences between AFRP and steel reinforcement.

The study by Xue et al. highlights that the bond mechanism between AFRP bars and concrete is fundamental to their structural application, with bond performance and failure modes strongly dependent on surface morphology [130]. The bond strength varies across different surface types, generally ranking in descending order as follows: braided > helical > ribbed > sand-coated > rough > plain-stranded. In contrast to steel reinforcement, where bond failure typically involves concrete crushing, failure in flexible braided AFRP bars is primarily governed by their interlaminar shear strength, often manifesting as severe surface abrasion or interlayer shear failure [129]. Additionally, the high Poisson’s ratio of AFRP leads to significant transverse contraction under tensile load, which reduces the radial confinement (i.e., wedging action) at the concrete interface and increases susceptibility to bond slip. Consequently, the bond stress of flexible braided AFRP bars generally peaks at relatively small slip displacements (4.5–10.5 mm), with the bond strength reaching approximately 70–90% of that of steel bars. At the structural member level, the linear-elastic behavior of flexible braided AFRP bars contributes to low ductility in prestressed beams. To enhance seismic performance, three effective technical measures have been proposed: (1) applying partial prestressing combined with non-prestressed steel reinforcement; (2) controlling the bond conditions of AFRP bars (e.g., using unbonded or partially bonded configurations); and (3) employing a hybrid system of internal and external prestressed tendons. Cyclic loading tests indicate that with these design strategies, AFRP prestressed beams can achieve a load-carrying capacity comparable to steel strand beams, attain displacement ductility coefficients in the range of 2.39–6.10, and exhibit improved residual deformation control [131]. However, due to the premature rupture of AFRP bars, their energy dissipation capacity remains lower than that of steel strand beams. Regarding theoretical modeling, current design codes show considerable deviations in predicting the bond strength of AFRP bars. A recently developed bond strength model, based on extensive experimental data, significantly enhances the prediction accuracy by incorporating key parameters such as surface morphology coefficient, specimen type coefficient, elastic modulus, and concrete cover thickness. This model provides an important tool for the refined design and safe implementation of AFRP-reinforced concrete structures. In related research, Takasago et al. [132] investigated the bond and cracking behavior of braided AFRP bars embedded in polyvinyl alcohol fiber-reinforced cementitious composites (PVA-FRCC). Pull-out tests demonstrated that the addition of polyvinyl alcohol fibers substantially improves both the bond strength and ductility between AFRP bars and FRCC, with bonding performance increasing alongside fiber volume fraction and member cross-sectional size. In flexural beam tests, AFRP-reinforced PVA-FRCC members exhibited multiple cracking patterns and enhanced ductility.

In summary, AFRP-reinforced concrete beams demonstrate a clear effect in improving load-bearing capacity. However, deformation control and brittle failure issues resulting from the low modulus of AFRPs require comprehensive solutions integrating prestressing techniques, optimization of bond conditions, and hybrid reinforcement strategies. While AFRP reinforcement is well-suited for applications demanding high deformation capacity or exposure to highly corrosive environments, its load-bearing efficiency remains comparatively lower than that of CFRP reinforcement. Consequently, achieving equivalent performance often necessitates a higher prestressing level. Nevertheless, key aspects remain insufficiently studied. The influence of AFRP surface morphology on bond behavior, along with the performance degradation mechanisms of AFRPs in alkaline concrete environments, has not been fully elucidated. Furthermore, a dedicated anchorage system designed to accommodate the mechanical properties of AFRP reinforcement has yet to be developed. Future research should therefore investigate in greater depth the combined effects of surface morphology, concrete properties, and testing methods on bond performance, which is essential for establishing accurate predictive models and reliable design guidelines. Additionally, the long-term performance degradation of AFRPs in alkaline environments, as well as their pronounced stress relaxation behavior in prestressed applications, must be thoroughly accounted for in the design of AFRP-reinforced concrete structures.

#### 3.2.2. Rock Anchor

Rock anchors are structural components in civil engineering designed to transfer tensile forces into stable underground rock or soil, thereby providing support and stabilization. Since their first application in 1934 for reinforcing the Cheurfas Dam in Algeria, ground anchors have become widely employed in various engineering contexts. These include slope stabilization, retaining walls, concrete dams, bridge abutments, and underground excavations. Additionally, they are frequently used to strengthen and repair existing structures, such as aging concrete dams. In mining engineering, ground anchors also serve as an essential support measure. Overall, their primary function lies in reinforcing and stabilizing structures including slopes, retaining walls, bridges, buildings, and dam bodies.

Traditional ground anchors typically employ prestressed steel in the form of strands or wires. However, steel anchors are susceptible to rust and corrosion in humid or chemically aggressive environments, which can significantly reduce their load-bearing capacity. Additionally, steel anchors may generate electromagnetic interference in the vicinity of sensitive equipment, potentially affecting its operation. The long-term performance of steel anchors is also influenced by factors such as corrosion and fatigue, necessitating regular inspection and maintenance. In contrast, FRP anchors present a potential alternative due to their high corrosion resistance, excellent tensile strength, low weight (approximately 15–20% that of steel), and superior fatigue performance. Their non-metallic nature also makes them compatible with embedded fiber optic sensors for structural health monitoring. Although the material cost of FRPs is generally higher than that of steel, their lightweight properties and elimination of corrosion protection requirements can lead to significant overall cost savings in transportation, handling, installation, and long-term maintenance. Specifically, AFRP bars exhibit not only high tensile strength, high elastic modulus, and strong durability in harsh environments, but also excellent resistance to out-of-plane shear. Similarly, under the protection of the internal matrix in AFRPs, the adverse effects of moisture and UV degradation on aramid fibers can be significantly mitigated. Therefore, AFRP bars exhibit not only high tensile strength, high elastic modulus, and strong durability in harsh environments, but also excellent resistance to out-of-plane shear. These characteristics make AFRPs suitable for use as permanent support anchors in civil engineering. Currently, several industrialized products are available, such as the CFRP anchor systems CFCC and Leadline produced in Japan, and the AFRP systems Arapree from the Netherlands, and FiBRA and Technora from Japan [133].

Zhang et al. [133] investigated the tensile and creep properties of AFRP and CFRP reinforcements when used as prestressed soil anchors. Their study demonstrated that CFRP anchors (e.g., Leadline and CFCC) exhibit a higher bearing capacity, smaller displacement, and superior long-term creep resistance, with a verification load reaching up to 0.83 times the ultimate load. In contrast, AFRP anchors (such as Arapree and Technora) showed larger slip and displacement, with failure primarily occurring due to interfacial debonding of the reinforcement. The bearing capacity of AFRP anchors was comparatively lower, with Arapree attaining only about 0.60 times the ultimate load. The study also noted that increasing the anchorage length significantly enhances the performance of CFRP anchors but has limited effect on AFRP anchors. Based on controlling creep performance for long-term safety, the authors recommended that the design working load for AFRP and CFRP anchors be limited to 0.40 and 0.50 times the ultimate load, respectively.

Brahim Benmokrane et al. [134] conducted a detailed comparative study on the mechanical, creep, and fatigue properties of CFRPs and AFRPs for anchor applications. Their work described the relevant anchoring systems and introduced practical engineering implementations. A key finding is that the transverse shear strength of composite anchors is significantly lower than their tensile strength, necessitating specialized anchorage design when using FRP materials. Furthermore, FRP anchors are susceptible to creep rupture under sustained loading, requiring designers to account for long-term strength reduction. In practice, CFRP (CFCC) anchors were employed in slope and retaining wall projects in Hokkaido in 1993, with design loads ranging from 92 kN to 510 kN and anchorage lengths of 3–7.5 m. Leadline anchors were used in 1990 for a pedestrian bridge abutment in Ibaraki Prefecture and in 1996 for a slope stabilization project in Fukuchiyama City. AFRP (Technora) anchors were applied in 1994 to a retaining wall on the Meishin Expressway and in 1995 to a slope along the Kawabe National Highway, with design loads of 400 kN and anchorage lengths of 3–6.5 m. These projects collectively demonstrate the feasibility of FRP anchors in design and construction. The advantages of AFRP anchors include corrosion resistance, long service life, ease of transportation and installation, and suitability for electromagnetically sensitive environments due to their non-magnetic properties. However, the development of dedicated anchoring systems for AFRPs is essential, and more field data are required to better understand their long-term performance limitations.

Although the feasibility of AFRP anchor bolts has been demonstrated in practical engineering, fundamental research on their performance remains relatively limited. Specifically, braided AFRP bars, which offer superior lateral shear resistance, show significant potential for applications in rock anchoring (e.g., in dam structures). However, studies on braided AFRP bars are still in the early stages, and further investigation is needed in several key areas. These include their manufacturing processes, testing and characterization of basic mechanical properties, as well as their long-term in-service performance.

## 4. Recommendations for Future Studies

Research on the application of AFRPs in civil engineering has predominantly concentrated on concrete reinforcement. Although sporadic studies and practical implementations exist in other civil engineering domains, several critical issues warrant further investigation:

(1) The primary constraint limiting the wider application of AFRPs in civil engineering is the poor interfacial bonding performance between aramid fibers and the resin matrix. Most existing interfacial modification methods, developed primarily under laboratory conditions, involve complex procedures, extended processing times, and high costs. Consequently, there is a pressing need to develop an interfacial modification technique that is simple to process, cost-effective, and amenable to continuous, large-scale industrial production.
(2) In the field of aramid fiber-reinforced concrete, considerable research has been conducted; however, the effective overlap length of aramid fiber sheets remains underexplored. Furthermore, while the influence of the number of wrapping layers has been examined, a systematic investigation is still needed to evaluate how parameters such as sheet thickness and the number of reinforcement layers affect the strengthening performance. Such research is essential to establish quantitative relationships between these parameters and key structural responses—including load-bearing capacity, ductility, and crack control—and to develop corresponding theoretical models.
(3) AFRPs can enhance the impact and blast resistance of concrete structures, with high-toughness variants offering particularly significant potential. However, the extent of improvement in the impact resistance of carbon fiber- and aramid fiber-reinforced concrete structures remains inconclusive, as it is influenced by multiple factors including fiber content, impact velocity, impact location, and angle of incidence. Furthermore, to establish a reliable predictive model for the impact resistance of AFRP-reinforced concrete, a key challenge that must be addressed is the development of appropriate test and characterization methods for the bond fracture energy between AFRP and concrete under varying strain rates.
(4) AFRPs exhibit superior transverse shear resistance, which helps address the anchoring challenges and large anchorage sizes typically encountered with CFRPs in prestressed applications. This makes them suitable for use in prestressed tendon-concrete structures. Nevertheless, in the design of AFRP–concrete systems, it is essential to fully account for the long-term performance degradation of AFRP tendons in the alkaline concrete environment, as well as the pronounced stress relaxation observed in AFRP tendons under prestressed conditions. Future research should quantify these degradation mechanisms and develop design guidelines that incorporate long-term effects.
(5) While the feasibility of AFRP anchor bolts has been confirmed in practical design and construction, fundamental research on their performance remains notably limited. Braided AFRP tendons show considerable promise for rock anchor applications; however, further investigation is required in several areas, including their manufacturing processes, characterization of basic mechanical properties, and long-term in-service performance, under different loading conditions, and the evaluation of long-term in-service performance including creep and environmental degradation.
(6) As a high-performance fiber, aramid fibers can be employed not only in the applications above-mentioned but also in specialized scenarios such as deep-sea tension-leg mooring systems and extraterrestrial construction. Prior to implementation in these emerging fields, systematic research is needed to establish comprehensive performance databases and design methodologies. Key knowledge gaps include the coupled effects of high hydrostatic pressure and seawater corrosion on AFRP mechanical properties, the influence of extreme temperature cycles and vacuum conditions on fatigue and creep behavior, and the development of reliable long-term durability prediction models tailored to these unique service environments.

## 5. Conclusions

This paper reviews the fundamental characteristics of AFRPs and its applications in civil engineering. The principal conclusions are as follows:

(1) Aramid fibers exhibit notable mechanical properties, including high tensile strength, temperature resistance, and chemical corrosion resistance. Influenced by their molecular structure, they demonstrate good tensile fatigue performance but poor compressive fatigue behavior. Within a suitable temperature range, aramid fibers are susceptible to short-term creep, while long-term creep is generally negligible. However, under elevated temperatures, their creep characteristics require particular attention. Additionally, aramid fibers possess low resistance to UV aging, with prolonged exposure leading to gradual degradation in strength. Therefore, appropriate UV protective treatments are necessary in practical applications.
(2) Surface treatments can enhance interfacial bonding between aramid fibers and the resin matrix; however, current methods are generally complex and remain primarily at the laboratory stage, limiting their large-scale engineering application. The development of simple, economical, and industrially scalable interfacial modification methods is crucial for advancing the use of AFRPs in civil engineering.
(3) The confinement effectiveness of AFRP-confined concrete columns is generally intermediate between that of CFRPs and GFRPs. These columns demonstrate balanced overall performance, particularly in confining square sections. Moreover, due to their inherent toughness and excellent corrosion resistance, AFRP-confined concrete columns exhibit favorable seismic performance and durability in high-temperature corrosive environments.
(4) Aramid fibers and their composite forms (e.g., fabric, sheet, rope, net, strip) significantly enhance the flexural, shear, and torsional capacity of concrete beams. The improvement in flexural strength typically lies between that provided by CFRPs and GFRPs, while AFRP strengthening often leads to more pronounced ductility. The effectiveness of the strengthening is influenced by multiple factors, including fiber content, bonding technique, substrate condition (e.g., corrosion), load type (static or fatigue), and reinforcement configuration. Consequently, the choice of strengthening method should be tailored to the specific application.
(5) AFRPs can effectively enhance the impact and blast resistance of concrete structures, and the bond performance between AFRPs and concrete plays a dominant role in governing failure mechanisms under dynamic loading.
(6) AFRP strengthening clearly improves the load-bearing capacity of concrete beams. The issues of deformation control and potential brittle failure associated with its lower modulus can be addressed through strategies such as prestressing, bond optimization, and hybrid reinforcement. AFRPs are particularly suitable for applications demanding high deformation capacity or situated in severely corrosive environments. While their load-bearing efficiency is typically lower than that of CFRPs, this can be compensated for by increasing the prestress level.
(7) Although the feasibility of AFRP anchor bolts has been validated in practice, fundamental research on their performance remains limited. The widespread application necessitates further systematic research into their mechanical, fatigue, creep, and long-term durability properties.

## Figures and Tables

**Table 1:** Comparison of properties of commonly used fibers in civil engineering.

Fiber	Type	Density (g/cm^3^)	Strength (MPa)	Modulus (GPa)	Elongation at Break (%)
Para-aramid [37]	DuPont: Kevlar-29	1.44	2900	71	3.6
	DuPont: Kevlar-49	1.44	3000	112	2.4
	Teijin: Twaron (stander)	1.44	2900	70	3.6
	Teijin: Twaron (High modulus)	1.44	2900	110	2.5
	Teijin: Technora (High strength)	1.39	3400	72	4.6
Carbon fiber [38]	T300	1.76	3500	230	1.5
	T700	1.8	4900	230	2.1
Glass fiber [39]	E-glass	2.5~2.7	3100~3800	76~80	4.5

**Table 2:** Test results of the aramid fiber-reinforced polymer composite-confined concrete columns.

Tensile Strength of Aramid Fiber (MPa)	Modulus of Aramid Fiber (GPa)	Ultimate Rupture Strain of Aramid Fiber (%)	Size of Columns	f_c_ (MPa)	Constraint Layers	f_cc_ (MPa)	f_cc_/f_c_	ε_c_	ε_cc_	ε_cc_/ε_c_	Ref	NO.
Cylinder columns												
2060	118	1.77	70 × 210	50.64	0.057 mm	65.97	1.30	0.244	0.403	1.65	[71]	(a)
			105 × 315		0.072 mm	59.48	1.17	0.244	0.331	1.36		
			194 × 582		0.143 mm	44.00	0.87	0.260	0.358	1.38		
			70 × 210	28.79	0.228 mm	86.07	2.99	0.202	0.953	4.72		
			105 × 315		0.191 mm	87.42	3.04	0.202	1.147	5.68		
			194 × 582		0.286 mm	80.86	2.81	0.207	0.933	4.51		
2060	118	1.77	100 × 300	46.43	0.286 mm	78.26	1.69	0.255	0.903	3.54	[72]	(b)
					0.572 mm	128.49	2.77	0.255	1.879	7.37		
				78.50	0.286 mm	118.33	1.51	0.451	1.082	2.40		
					0.572 mm	167.05	2.13	0.451	1.424	3.16		
					0.858 mm	185.78	2.37	0.451	1.611	3.57		
				101.18	0.286 mm	123.27	1.22	0.456	0.627	1.38		
					0.572 mm	153.95	1.52	0.456	1.016	2.23		
					0.858 mm	204.51	2.02	0.456	1.437	3.15		
2060	118	1.75	150 × 450	47.3	1 (0.572 mm)	81.54	1.72	0.267	1.40	5.24	[73]	(c)
			150 × 450	51.1	1 (0.572 mm)	83.76	1.64	0.267	1.39	5.21		
3732	115.2	3.24	152 × 305	39.2	1 (0.169 mm)	59.97	1.53	0.233	0.316	1.36	[74]	(d)
					2 (0.338 mm)	87.23	2.23	0.233	0.297	1.27		
					3 (0.507 mm)	115.83	2.95	0.233	0.272	1.17		
2390	128.5	1.86	63 × 126	51.6	1 (0.2 mm)	104.4	2.02	/	0.287	/	[69]	(e)
				51.6	2 (0.4 mm)	168.5	3.27	/	0.444	/		
				128	2 (0.4 mm)	148.57	1.16	/	0.170	/		
2900	116	2.50	152 × 305	49.4	3 (0.6 mm)	106.27	2.15	0.24	0.339	1.41	[65]	(f)
2900	120	2.50	152.5 × 305	39	2 (0.4 mm)	68.15	1.75	0.21	2.31	11.00	[75]	(g)
				39	3 (0.6 mm)	86.3	2.21	0.21	2.985	14.21		
				101	4 (0.8 mm)	120.5	1.19	0.34	1.37	4.03		
				106	6 (1.2 mm)	153.95	1.45	0.35	1.70	4.86		
2390	128.5	1.86	152.5 × 305	85.7	6 (1.2 mm)	166.47	1.94	0.24	2.10	8.75	[76]	(h)
				112.4	6 (1.2 mm)	165.67	1.47	0.27	1.86	6.89		
				120.9	6 (1.2 mm)	169.2	1.40	0.26	1.77	6.81		
				113.5	6 (1.2 mm)	178.57	1.57	0.26	1.94	7.46		
2188.5	128.5	3.60	100 × 200	34.4	1 (not give)	44.0	1.28	0.2	1.21	6.05	[77]	(i)
					2 (not give)	65.9	1.92	0.2	1.82	9.10		
					3 (not give)	80.7	2.35	0.2	2.18	10.90		
				21.0	1 (not give)	42.3	2.01	0.2	1.58	7.90		
					2 (not give)	59.5	2.83	0.2	2.20	11.00		
					3 (not give)	75.3	3.59	0.2	2.74	13.70		
			150 × 300	33.1	1 (not give)	41.7	1.26	0.2	1.94	9.70		
					2 (not give)	53.2	1.61	0.2	1.96	9.80		
					3 (not give)	66	1.99	0.2	1.86	9.30		
				21.3	1 (not give)	35.0	1.64	0.2	2.19	10.95		
					2 (not give)	46.8	2.20	0.2	2.30	11.50		
					3 (not give)	61.8	2.90	0.2	1.85	9.25		
2400	122	2.06	100 × 200	74.5	1 (0.185 mm)	85.0	1.14	0.225	1.48	6.58	[66]	(j)
					2 (0.37 mm)	114.0	1.53	0.225	1.66	7.38		
					3 (0.555 mm)	140.2	1.88	0.225	2.07	9.20		
2010	107.6	2.10	150 × 300	20.9	1 (0.288 mm)	54.1	2.59	0.19	2.15	11.32	[78]	(k)
			150 × 300	52.7	1 (0.288 mm)	57.5	1.09	0.21	0.91	4.33		
			150 × 300	52.7	2 (0.576 mm)	84.7	1.61	0.21	1.865	8.88		
			150 × 300	110.6	1 (0.288 mm)	93.1	0.84	0.26	1.14	4.38		
2059	118	1.75	76 × 305	44	2 (0.872 mm)	150.5	3.42	0.19	2.05	10.79	[79]	(l)
Square Column												
2060	118	1.77	70 × 210	34.61	0.0715 mm	44.40	1.28	0.273	0.632	2.32	[71]	(m)
			100 × 300	34.61	0.0953 mm	41.01	1.18	0.273	0.475	1.74		
			150 × 450	34.61	0.1430 mm	32.99	0.95	0.19	0.590	3.11		
			70 × 210	34.61	0.2860 mm	86.20	2.49	0.219	0.658	3.00		
			100 × 300	34.61	0.3814 mm	76.32	2.21	0.219	0.524	2.39		
			150 × 450	34.61	0.5720 mm	54.27	1.57	0.213	0.544	2.55		
2188.5	128.5	3.6	100 × 200	33.1	1 (not give)	41.0	1.24	0.2	1.08	5.40	[77]	(n)
					2 (not give)	51.5	1.56	0.2	1.66	8.30		
					3 (not give)	62.2	1.88	0.2	2.23	11.15		
				24.4	1 (not give)	34.0	1.39	0.2	1.23	6.15		
					2 (not give)	43.9	1.80	0.2	1.77	8.85		
					3 (not give)	58.3	2.39	0.2	2.36	11.80		
2400	122	2.06	100 × 200	74.5	1 (0.185 mm)	62.25	0.84	0.225	0.76	3.38	[66]	(o)
					2 (0.37 mm)	75.0	1.01	0.225	1.26	5.60		
					3 (0.555 mm)	99.7	1.34	0.225	1.73	7.69		

## Data Availability

No new data were created or analyzed in this study. Data sharing is not applicable to this article.
